# Absolute quantification of tumor necrosis factor-alpha by isotope dilution mass spectrometry

**DOI:** 10.3389/fchem.2025.1667885

**Published:** 2026-02-06

**Authors:** Chenchen Bao, Jing Wang, Wenqiang Xia, Ke Yang, Xinchang Shi, Xiaoying Yang, Hailin Li, Yuemin Wang, Yafen Zhang

**Affiliations:** 1 Zhejiang Provincial Key Laboratory of Biometrology and Inspection & Quarantine, College of Life Sciences, China Jiliang University, Hangzhou, China; 2 Institute of Crop Science, College of Agriculture and Biotechnology, Zhejiang University, Hangzhou, China; 3 NMPA Key Laboratory for Quality Research and Evaluation of Biological Products, National Institute for Food and Drug Control, Beijing, China

**Keywords:** amino acid, isotope dilution mass spectrometry, peptide segment, quantification, tumor necrosis factor-α

## Abstract

Tumor necrosis factor-α (TNF-α) is an important inflammatory mediator and has been widely recognized as a diagnostic biomarker for various autoimmune and infectious diseases in clinical practice, such as rheumatoid arthritis. In this study, we established an SI-traceable reference method to quantify TNF-α based on liquid chromatography–isotope dilution tandem mass spectrometry (LC-IDMS) with amino acid or peptide analysis. The assays exhibited good linearity (*R* ^2^ > 0.999), repeatability (RSD < 3%) and accuracy, which had been verified using certified reference materials (CRMs). Stable isotope-labeled version of three amino acids (valine, phenylalanine, and leucine) and two peptides were used as an internal standard to minimize assay variability. For amino acid analysis, TNF-α could be fully hydrolyzed into amino acids after 60 h at 110 °C. The result based on the amino acid analysis was (0.770 ± 0.033) mg/g, expressed as a mass fraction (mg TNF-α per g of total solution), with an expanded uncertainty (*k* = 2). For peptide analysis, ANALLANGVELR (AR-12) and VVNLLSAIK (VK-9) were chosen as specific peptides. After 36 h of tryptic proteolysis, TNF-α could be completely proteolyzed into AR-12 and VK-9. Based on characteristic peptide analysis, the result was 0.769 ± 0.046 mg/g (*k = 2*). There was no significant difference between these two analyses, and the concentration of TNF-α was 0.770 ± 0.057 mg/g (*k = 2*), which was traceable to the International System of Units. Both methods developed in this study can accurately determine the concentration of TNF-α and are useful for detection kit development and instrument calibration. In addition, application data for reagents certified by these methods in cell apoptosis assays and kit evaluation are provided: TNF-α-induced cell apoptosis was significantly attenuated by antagonists, while detection kits based on three different principles exhibited good repeatability (RSD < 9%) and linearity (*R* ^2^ > 0.999). This accurate, SI-traceable method can improve clinical TNF-α assay standardization and biomarker reliability.

## 1 Introduction

Tumor necrosis factor-α (TNF-α) is the most potent anti-tumor cytokine discovered so far (De La Cruz-Vargas et al., 2025), exhibiting a wide range of biological activities. TNF-α can promote the production and secretion of IL-1, IL-2, and IL-6 through the secretion of immune cells, such as activated T cells and macrophages (Balkwill, 2009); induce inflammatory responses; and promote the expressions of IL-2R, EGFR, and the major histocompatibility antigen class of antigens (MHC IIAg) and other expressions, forming a complex immune network that plays an important role in the host defense response (Yang et al., 2022). It has been found that TNF-α plays a mediating role in Crohn’s disease (Gisbert and Chaparro, 2023; Veerasubramanian et al., 2024), rheumatoid arthritis (Hu et al., 2024; Sorokin and Mehta, 2022), psoriasis (Sun et al., 2024; Wride et al., 2024), tumor proliferation and differentiation, and inhibition of apoptosis (Jang et al., 2021; Tiegs and Horst, 2022). Therefore, the accurate determination of TNF-α is important for the early screening and prognosis of diseases. To ensure the traceability, accuracy and comparability of the measurement results and achieve standardization of clinical testing, it is necessary to establish accurate and reliable reference methods to meet the needs of clinical testing.

Liquid chromatography–isotope dilution tandem mass spectrometry (LC-IDMS/MS) has become an important tool for comprehensive characterization and quantification of proteins in the field of biochemistry, due to its specificity, accuracy, and sensitivity and serves as a reference method for numerous clinical indicators (Deng et al., 2023; Liu et al., 2021). Targeted proteomics technology involves the enzymatic hydrolysis of proteins into amino acids by acid or into peptides, which are then analyzed through liquid chromatography–tandem mass spectrometry (Aebersold and Mann, 2003).

Amino acid-based isotope dilution mass spectrometry (AA-IDMS) markedly improves the precision of the assay due to the inclusion of stable isotopes of known mass and abundance in the pretreatment, which reduces the interference of random and systematic errors. For the amino acid-based IDMS method, using internally standardized amino acid isotopes such as valine (Val), phenylalanine (Phe), and leucine (Leu), which are chemically stable at high temperatures in acidic solutions, the hydrolysis conditions, including hydrolysis time, should be optimized to ensure complete hydrolysis of amino acids in the protein.

For the IDMS method based on characteristic peptide fragments (PEP-IDMS), screening the characteristic peptide fragments in combination with the amino acid sequence of target proteins and realizing the qualitative and quantitative analyses of target proteins by detecting the characteristic peptide fragments possess the advantages of high sensitivity and good stability and have now become a commonly used method for qualitative and quantitative analyses of proteins. Peptide map sample processing involves protein denaturation, disulfide bond reduction, alkylation closure, enzymatic cleavage, etc. Since salts, unreacted enzymes, and impurities generated during enzymatic cleavage may interfere with the sensitivity and accuracy of the assay, desalting after enzymatic cleavage can lead to better reproducibility of the samples and improve the ionization efficiency of peptides (Bitter et al., 2023). Several targeted and untargeted proteomics studies have evaluated the quantitative response of trypsin and described the rules that guide peptide selection for protein quantification (Lange et al., 2008; Searle et al., 2015; Worboys et al., 2014). Trypsin recognizes lysine K and arginine R. The peptide fragments obtained after proteolytic cleavage provide better sequence coverage, and trypsin is the most commonly used enzyme in mass spectrometry-based proteomics experiments due to its high specificity, wide availability, and ease of use (Sun et al., 2023).

**FIGURE 1:**
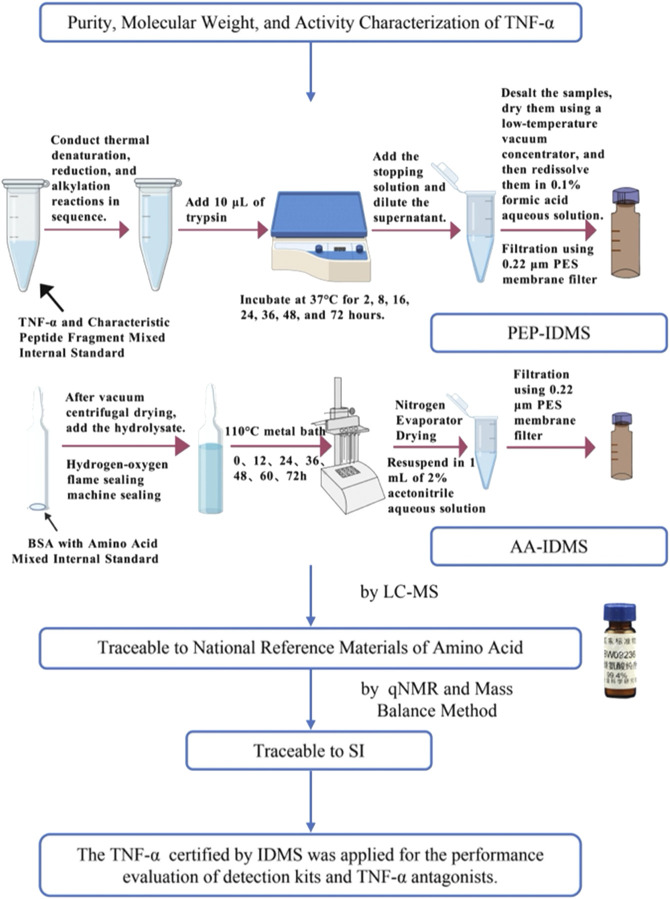
Traceability chains of the two detection methods. Created with BioGDP.com (Jiang et al., 2025).

We here investigated the quantification method of recombinantly expressed TNF-α protein using two different protein quantification methods. The first method involves hydrolyzing TNF-α to amino acids with acid, determine the amino acid concentration by mass spectrometry, and verify the reliability of the assay using bovine serum albumin (BSA) standards. The second method involves using IDMS based on peptide analysis, synthesize the screened characteristic peptides, use IDMS based on amino acids determine the absolute concentration of the target standard peptides, optimize the conditions for enzymatic digestion of TNF-α, and finally determine the peptide concentrations using the optimal conditions. Currently, IDMS has become a powerful tool for biomedical research and clinical proteomics owing to its high sensitivity, quantitative accuracy, and reproducibility (Chen and Liu, 2018), as exemplified by its successful application in the SI-traceable quantification of other critical biomarkers, e.g., C-peptide (Cho et al., 2025). To our knowledge, however, there is no study on quantitative analysis of TNF-α by acid hydrolysis and peptide digestion. Accurate quantification of TNF-α is of great significance for disease diagnosis and monitoring, drug discovery and development, etc. However, the limitations including many TNF-α detection methods, sample stability and drug interference seriously affect the accurate quantification of TNF-α (Farney et al., 2011). To address these issues, we include two quantification methods that exhibit high specificity and sensitivity, can reduce the sample processing error, and are traceable to the International System of Units (Figure 1). Therefore, the present study is helpful for the absolute quantification of TNF-α protein and the development of related reagents and drugs.

## 2 Materials and methods

### 2.1 Instruments and devices

Ultra-high-performance liquid chromatography–triple quadrupole mass spectrometry was performed using an ACQUITY UPLC I-Class system coupled to a Xevo TQ-XS mass spectrometer (Waters, United States), and sample weights were measured using a precision electronic balance (XSR105, Mettler Toledo, Switzerland).

### 2.2 Materials and reagents

L-leucine (Leu, 99.7%, GBW 09237), L-phenylalanine (Phe, 99.8%, GBW 09235), L-valine (Val, 99.4%, GBW 09236), and BSA standards (NIM-RM3627-9) were purchased from the China Academy of Metrology. Isotopically labeled amino acids including ^13^C_6_-leucine (Leu*, 99%, CNLM-281-H-0.05), ^13^C_9_-phenylalanine (Phe*, 99%, CNLM-575-H-0.1), and ^13^C_5_-valine (Val*, 99%, CNLM-442-H-0.25) were purchased from Cambridge Isotope Laboratories, United States. Characteristic peptides and their isotope-labeled peptides, such as ANALLANGVELR (P230925-SJ398866), VNLLSAIK (P230925-SJ1110671), ANALLA (^13^C_3_,^15^N)NGVELR (P240701-LL1179987), and VNLLSA (^13^C_3_,^15^N)IK (P240701-LL1179986), were purchased from Shanghai Gill Biochemical Co. Trypsin (sequencing grade-modified trypsin, v5111) was purchased from Promega, Madison, WI, United States. RapiGest SF (186001861) was purchased from Waters. Dithiothreitol (DTT, 98%, 43815) and iodoacetamide (IAM, 99%, I1149) were purchased from Sigma, Germany. Formic acid (FA, LC-MS grade, 942988) was purchased from J&K Scientific, China. Trifluoroacetic acid (TFA, LCMS grade, 85183) was purchased from Thermo, Waltham, MA, United States. Acetonitrile (LC-MS grade, A120771) was obtained from Aladdin, China. Hydrochloric acid (HCl, 36%, AR, P1666227) was obtained from Shanghai Titan Technology Co. Mouse connective tissue L cell line 929 clonal cells (CL0339) were purchased from Fung Fai Bio, China. The human hepatoma cell line HepG2 (TCH-C196) was purchased from HyCyte, China. Actinomycin D (HY-17559) and TNF-α antagonist (R-7050, HY-110203, MedChemExpress) were purchased from MedChemExpress, New Jersey, United States.

### 2.3 Experimental solution preparation

TNF-α was diluted to approximately 0.8 mg/mL using a diluent (150 mM NaCl, 0.5% alginate, and 20 mM Tris HCl; pH 8.0) and stored at −80 °C. The concentration is expressed as a mass fraction (mg TNF-α per g of total solution); for this aqueous solution, it is numerically equivalent to mg/mL and ensures SI traceability through gravimetric preparation.

The mixed amino acids were accurately weighed to a mass close to the mass of amino acids in the hydrolyzed TNF-α. Mixed characteristic peptides were also prepared according to the peptides in tryptic proteolyzed TNF-α. Low-standard solution: the mass ratio of mixed labeled amino acids (L-AAs) or labeled characteristic peptides (L-PEPs) to mixed standard amino acids (S-AAs) or standard characteristic peptides (S-PEPs) was 0.9. High-standard solution: the mass ratio of mixed L-AAs/L-PEPs to mixed S-AAs/S-PEPs was 1.1.

### 2.4 Characterization of TNF-α purity

The purity of TNF-α was analyzed using high-performance liquid chromatography (HPLC) and SDS-PAGE.

Size exclusion chromatography was performed with an injection volume of 20 μL at a flow rate of 0.2 mL/min using mobile phase A containing 50 mM phosphate and 300 mM NaCl (pH 6.8), with an mobile phase program of 100% A from 0 to 30 min. It was performed using an Mabpac SEC-1 column (300 Å, 5 μm, 4.0 × 300 mm), detection carried out using an HPLC detector (Acquity Arc, Waters).

SDS-PAGE: After heating and denaturation, TNF-α with proportional protein uploading buffer was spiked into the gel and separated at 100 V. The gel was stained with Coomassie Brilliant Blue and analyzed using the Bio-Rad Gel Doc XR + gel imaging system.

### 2.5 Characterization of molecular weight

Quadruple-stage rod-time-of-flight mass spectrometry (XevoG2-XS Q-TOF, Waters) detection was performed by acidified dilution of the monomeric TNF-α solution with 1% formic acid aqueous solution at a 1:10 volume ratio, followed by peptidyl-N-glycosidase F digestion to release sugar chains, desalting treatment, and re-dissolution of the solution using acidified acetonitrile. The separation was carried out on a BioResolveTM RP mAb Polypheyl (450 Å, 2.7 μm, 2.1 mm × 50 mm, Waters) column in an ultra-high-performance liquid chromatography system (Acquity UPLC I-Class, Waters), with 0.1% formic acid aqueous solution used as the mobile phase A and 0.1% formic acid acetonitrile solution as the mobile phase B, the flow rate was 0.3 mL/min and the mobile phase program was 0–1 min: 75% A: 25% B; 7 min: 55% A: 45% B; 7.5 min: 10% A: 90% B; 7.6 min: 75% A: 25% B; 8.0 min: 10% A: 90% B; 8.10–10 min: 75% A: 25% B. Detection was performed in the positive-ion mode, and the scanning range of parent ions was 500–4,000 m/z. Raw data were processed using UNIFI (1.9.4, Waters) software.

Matrix-assisted laser desorption-tandem time-of-flight mass spectrometry (MALDI-TOF) detection was also used for molecular weight identification of TNF-α. The protein was mixed with the matrix auxiliary solution (acetonitrile solution containing 50 mg/mL of hydroxycinnamic acid). After the sample was crystallized, it was detected using MALDI-TOF. Positive polarity analysis was performed under pulsed ion extraction at 450 ns. The high voltages of ion sources 1, 2, and lenses were 20, 18.4, and 6.2 kV, respectively. FlexAnalysis (Bruker, United States) software was used to process the data.

### 2.6 Bioactivity analysis of TNF-α

Bioactivity analysis of TNF-α was performed using surface plasmon resonance (SPR), detection kits, and TNF-α-induced cell apoptosis assay.

SPR experiments were performed using a Biacore 8K (Cytiva, United States) system. Human TNFR1 was diluted to a working concentration of 5 μg/mL using a sodium acetate buffer system (10 mM, pH 4.5). Carboxyl group activation on the surface of the CM5 chip was carried out using an amino coupling method with an equimolar mixture of EDC/NHS. TNF-α was formulated with phosphate buffer containing 0.05% Tween-20 and was subjected to serial dilutions to establish multiple concentration gradients (250 nM–15.625 nM). Glycine–HCl was used as the regeneration solution for chip surface treatment.

The raw sensograms were processed using a double-referencing method, subtracting signals from both an unmodified reference flow cell and blank buffer injections. The corrected data were analyzed using Biacore Insight Evaluation Software. Binding curves from all analyte concentrations were globally fitted to a 1:1 Langmuir binding kinetic model to obtain the association rate constant (ka) and dissociation rate constant (kd). The equilibrium dissociation constant (KD) was calculated from the ratio of the dissociation and association rate constants (kd and ka).

A series of TNF-α solutions with known concentrations were used to evaluate the reproducibility and linearity of three test kits based on different detection principles (magnetic particulate chemiluminescence, chemiluminescence, and flow cytometry). Each experiment was repeated at least three times to ensure data reliability.

The biological activity of TNF-α was assessed in HepG2 and L929 cells seeded at 2 × 10^4^ cells/well. After 24 h of co-incubation with TNF-α (plus 1 μg/mL actinomycin D) and/or a small-molecule TNF-α inhibitor (R-7050), a working concentration of 5 μM/mL was used for the functional assays. Cell morphology was then examined. Viability was determined using the CCK-8 assay, measuring the absorbance at 450 nm.

### 2.7 Selection of characteristic peptides

Under the action of trypsin, TNF-α is cleaved at its specific sites, generating multiple characteristic peptides. By collecting signals from the enzymatic digestion products and comparing them with peptide signals in the database, the system achieved accurate identification of the target protein. After multiple digestion experiments, the system screened two out of six characteristic peptides that met the following criteria: good reproducibility, high mass spectrometric sensitivity, intact retention of cleavage sites, appropriate sequence length, and inclusion of at least one stable isotope tag.

The two standard peptides selected for quantitative analysis lacked certified reference materials (CRMs). AA-IDMS was used in this study for precise quantification, effectively establishing a metrological traceability chain of the assay data.

### 2.8 AA-IDMS

Equal amounts of TNF-α and mixed amino acids were weighed into an ampoule, and the ampoule was sealed after adding hydrochloric acid. Complete acid hydrolysis was achieved by incubation at 110 °C for 60 h (optimized via a time-course study of 0–84 h). The sample was then dried, re-dissolved, and filtered through a 0.22-μm membrane.

### 2.9 PEP-IDMS

Equal amounts of TNF-α and mixed peptides were weighed into a centrifuge tube. To disrupt the protein structure, Rapigest SF was added for heat denaturation, followed by the addition of DTT and IAM to perform reduction and alkylation reactions, ensuring complete unfolding of the target protein. Trypsin was added, and the mixture was incubated at 37 °C. The optimal digestion duration was determined to be 36 h based on a time-course experiment (2–72 h). The enzyme reaction was terminated by adding trifluoroacetic acid. After being desalted, dried, re-dissolved, and filtered, the sample was ready for detection.

### 2.10 Methodological verification

Linear analysis: L-AAs/L-PEPs and S-AAs/S-PEPs were weighed, with different concentration gradients. A standard curve was established by taking the ratio of the peak area between standard materials and isotope-labeled materials as the horizontal coordinate, with the mass ratio between them serving as the vertical coordinate.

Precision analysis: Three mass fractions (high, medium and low) of TNF-α samples were prepared. Three samples were measured per day for each concentration for 3 consecutive days, and the intra- and inter-day precisions were calculated.

Accuracy analysis: The accuracy of AA-IDMS was evaluated using CRM. Detection results of TNF-α content based on AA-IDMS and PEP-IDMS were statistically analyzed.

### 2.11 Calculation of results

The content of TNF-α standard material candidates was calculated as shown in Equations 1, 2.

$$w_{TNF}=\frac{w_{ts}}{{Mr}_{ts}\cdot Z_{ts}}\times {Mr}_{TNF}\times P_{TNF,}$$ (1)

$$w_{ts}=\left[\frac{\frac{m_{ref\;\left(high\right)}}{m_{iso\;\left(high\right)}}\;‐\;\frac{m_{ref\;\left(low\right)}}{m_{iso\;\left(low\right)}}}{\left(R_{high}\;‐\;R_{low}\right)}\cdot \left(R_{sam}‐R_{low}\right)+\frac{m_{ref\;\left(low\right)}}{m_{iso\;\left(low\right)}}\right]\cdot \frac{m_{iso}}{m_{sample}}\cdot \frac{m_{crm}}{m_{crm}+m_{s}}\cdot w_{ts,}$$ (2)

where

$w_{TNF}$ = mass fraction of TNF-α in the candidate;

$w_{ts}$ = mass fraction of the target substance in the candidate;

${Mr}_{ts}$ = relative molecular mass of the target substance;

${Mr}_{TNF}$ = relative molecular mass of TNF-α;

$Z_{ts}$ = number of the target substance in TNF-α;

$P_{TNF}$ = purity of TNF-α;

$m_{ref\;\left(high\right)}$ = mass of the CRM added in the high-standard solution;

$m_{iso\;\left(high\right)}$ = mass of the isotopic internal standard added in the high-standard solution;

$m_{ref\;\left(low\right)}$ = mass of the CRM added in the low-standard solution;

$m_{iso\;\left(low\right)}$ = mass of the isotopic internal standard added in the low-standard solution;

$R_{high}$ = peak area ratio of the target substance to its isotopic internal standard in the high standard;

$R_{low}$ = peak area ratio of the target substance to its isotopic internal standard in the low standard;

$R_{sam}$ = peak area ratio of the target substance to its isotopic internal standard in the sample;

$m_{iso}$ = mass of the isotopic internal standard added in the sample;

$m_{sample}$ = mass of the TNF-α added in the sample;

$m_{crm}$ = mass of the target substance added during the preparation of the mixed target substances;

$m_{s}$ = mass of the solvent added during the preparation of the mixed target substances;

$w_{ts}$ = content of the target substance in the target substance working solution.

The equivalence was calculated as shown in Equation 3.

$$E_{n}=\frac{\left|y‐y_{s}\right|}{\sqrt{U_{TNF}^{2}‐U_{s}^{2}}},$$ (3)

where

*y*, Quantitative result based on amino acids

*y*_*s*_, Concentration as shown in the NSS certificate

*u*, Extended uncertainty of the assay result

*U*_*s*_, Extended uncertainty as shown in the NSS certificate

### 2.12 LC-MS quantification conditions

A Waters CORTECS UPLC T3 (1.6 μm 2.1 × 150 mm) column was used for amino acid analysis. Mobile phases: 0.1% formic acid aqueous solution in phase A, 0.1% formic acid acetonitrile solution in phase B; program: 0–11 min: 98% A: 2% B; injection volume: 2 μL; flow rate: 0.3 mL/min.

A Waters CORTECS T3 (2.7 μm 2.1 × 150 mm) column was used for peptide analysis. Mobile phases: 0.1% formic acid aqueous solution in phase A, 0.1% formic acid acetonitrile solution in phase B. Program: 0 min: 95% A: 5% B; 4 min: 65% A: 35% B; 6 min: 0% A: 100% B; 7–10 min: 95% A: 5% B; injection volume: 2 μL; flow rate: 0.3 mL/min.

Amino acid analysis mass spectrometry conditions: The MRM mode was used to monitor ion pairs; leucine quantitative ion pair: (132.09→86.16); leucine internal standard quantitative ion pair: (139.03→92.15); phenylalanine quantitative ion pair: (165.97→120.10); phenylalanine internal standard quantitative ion pair: (176.03→129.07); valine quantitative ion pair: (117.97→72.06); valine internal standard quantitative ion pair: (123.97→77.07).

Peptide analysis mass spectrometry conditions: The MRM mode was used to monitor the ion pairs: candidate characteristic peptide quantitative ion pairs: ANALLANGVELR (AR-12): (620.7→758.5); VNLLSAIK (VK-9): (429.45→644.5); IS-ANALLANGVELR: (623.15→762.5); IS-VNLLSAIK: (431.4→648.5).

## 3 Results and discussion

### 3.1 Characterization of protein purity

**FIGURE 2:**
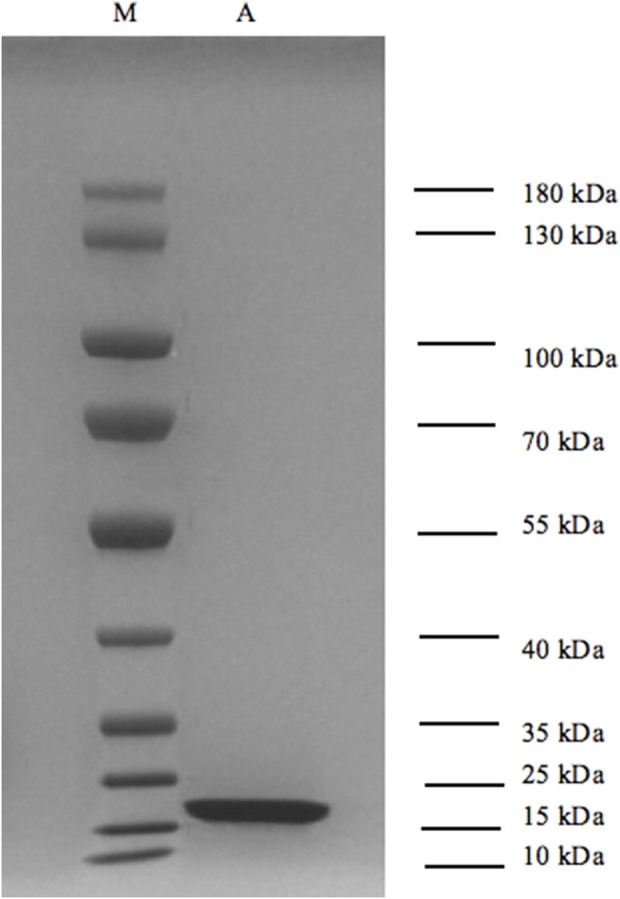
Results of SDS-PAGE electrophoresis of TNF-α protein. (A) TNF-α; (M) molecular weight markers.

As shown in Figure 2, by comparison with the marker, the molecular weight of the sample was observed to be 15–20 kDa, and no obvious impurity bands were detected, indicating high protein purity.

As shown in Supplementary Figure S1A, the main peak of the TNF-α protein appeared at 13.0 min through SEC column detection. The purity of TNF-α calculated using the area normalization method was 99.44%, 99.52%, and 99.94%, with an average value of 99.63% and relative standard deviation (RSD) of 0.68%.

### 3.2 Characterization of the molecular weight

Using Expasy’s ProtParam tool, a protein relative molecular weight prediction software tool, the amino acid sequence of TNF-α was analyzed, and the theoretical molecular weight was calculated to be 17.66 kDa, which was basically consistent with the SDS-PAGE results. Therefore, the separation and molecular weight detections of TNF-α were first performed using HPLC and quadrupole time-of-flight tandem mass spectrometry. The detection results are shown in Supplementary Figure S2.

As shown in Supplementary Figure S2, the relative molecular weight of TNF-α protein after glycosylation was 18.07 kDa, which was basically consistent with the theoretical value. There are also peaks at 17.90 kDa, 18.06 kDa, and 18.78 kDa shown in the figure, and it was hypothesized that the detected molecular weight deviation was induced by incomplete glycosylation. Human TNF undergoes O-glycosylation modification on Ser80 but not N-glycosylation (Takakura-Yamamoto et al., 1996), and O-glycosylation occurs in the Golgi apparatus, forming O-glycosidic bonds that serve as a linkage to the serine, threonine, hydroxylysine, and hydroxyproline hydroxyl groups, which are more direct and less homogeneous than N-glycosylation (Murakami et al., 2024).

The original molecular weight of TNF-α before glycosylation was detected using MALDI-TOF, and a main peak at 18.77 kDa was detected in the sample as the main component TNF-α, as shown in Supplementary Figure S3.

Glycosylation results in an inhomogeneous molecular weight of TNF-α, and the average molecular weight of TNF-α was calculated according to Equation 4:

$$MW=\frac{\sum \left(m/z\times Intensity\right)}{\sum Intensity}-H^{+}.$$ (4)

The measurements were repeated three times, and the average molecular weights of TNF-α were 18769.313 Da, 18773.465 Da, and 18773.624 Da. The results of MALDI-TOF, SDS-PAGE and Q-TOF were corroborated with each other. The average molecular weight of TNF-α was 18772.134 Da.

### 3.3 Bioactivity analysis of TNF-α

#### 3.3.1 SPR

As shown in Supplementary Figure S4, the adsorption between TNF-α and human TNFR1 reached equilibrium in approximately 1 min. The SPR response signal increased with increasing concentrations of TNF-α. Human TNFR1 (5 μg/mL) exhibited a good linear relationship with concentrations of TNF-α ranging from 15.625 nM to 250 nM. As shown in Table 1, the dissociation constant (KD) between TNF-α and human TNFR1 was 1.91 × 10^−9^ M. It was shown that the affinity constant for cell surface receptor–ligand binding (10^−8^-10^−10^ M) was larger than that for antigen–antibody interaction (10^−5^-10^−7^ M), and the affinity constant of TNF with its monoclonal antibody KD was 1.03 × 10^−7^ M (Yang et al., 2002). It indicates that the TNF-α sample used in this study exhibited good biological activity.

**TABLE 1:** TNF-α/human TNFR1 Kd values obtained by SPR measurements.

Interaction pair	Ka (M^–1^s^–1^)	Kd (s^–1^)	KD (M)	Rmax (RU)	Kinetics Chi^2^ (RU^2^)
TNF-α/human TNFR1	2.88 × 10^5^	5.49 × 10^−4^	1.91 × 10^−9^	49.4	1.46

#### 3.3.2 TNF-α detection kits

Different concentrations of TNF-α were measured using magnetic particle chemiluminescence assay kits. The theoretical concentration was linearly fitted to the detection results of the kits, as shown in Supplementary Figure S5, and the correlation coefficient (*R* ^2^) was 0.9991, showing ideal linear results. In addition, the detection results of chemiluminescence and flow fluorescence showed a good linear relationship, with *R* ^2^ values of 0.9996 and 0.9999, respectively.

Three parallel assays were performed for different concentrations of TNF-α, and the data are shown in Supplementary Table S1. The reproducibility of three detection systems—magnetic particle chemiluminescence assay, chemiluminescence assay and flow fluorescence assay—was evaluated, with RSDs ranging from 1.3% to 1.7%, 2.6% to 8.9%, and 0.9% to 3.2%, respectively. These data demonstrated that all tested kits possessed satisfactory reproducibility, thus meeting quantitative analysis requirements. The chemiluminescent kit showed slightly more variability at the low end of the range, possibly due to its detection chemistry.

#### 3.3.3 TNF-α-induced apoptosis in HepG2 cells

**FIGURE 3:**
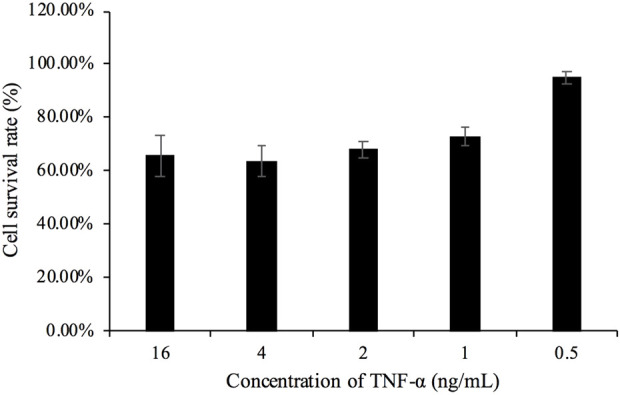
Cell activity induced by different doses of TNF-α and actinomycin D.

As shown in Figure 3, the cell survival rate significantly decreased at a TNF-α concentration of 1 ng/mL and was 64% at a TNF-α concentration of 4 ng/mL. The concentration of TNF-α was derived from traceable certified value results in Section 3.5.6.

**FIGURE 4:**
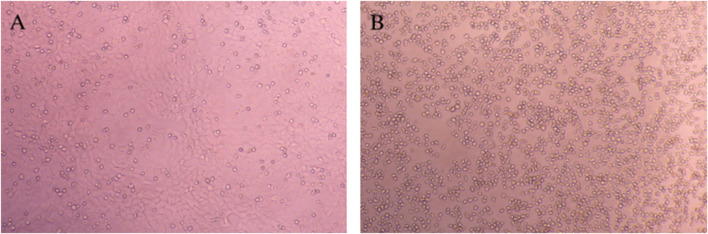
Morphological characteristics of HepG2 cells. **(A)** Blank control group; **(B)** TNF-α combined with the actinomycin D stimulation group.

As shown in Figure 4A, the control group exhibited uniform cell distribution, moderate density, and normal cell morphology. In Figure 4B, the cell density in the group of TNF-α combined with actinomycin D was observed to be significantly reduced, with contracting and aggregating cells, which was consistent with the ability of high-dose TNF-α to promote cell apoptosis (Ma et al., 2008).

#### 3.3.4 TNF-α antagonists inhibit TNF-α-induced apoptosis of L929 cells

**FIGURE 5:**
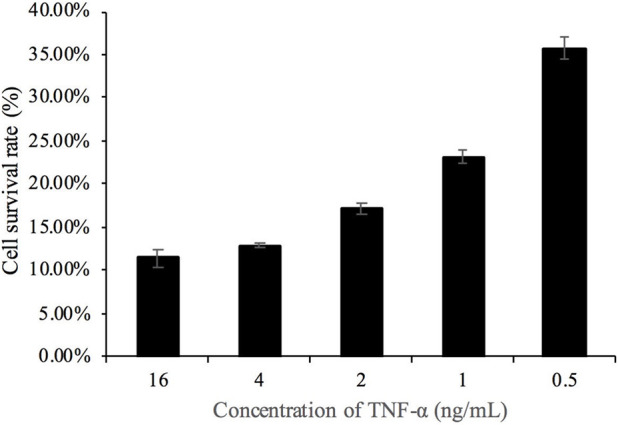
Concentration screening of TNF-α-induced cell death. Note: Contains 1 μg/mL actinomycin D.

**FIGURE 6:**
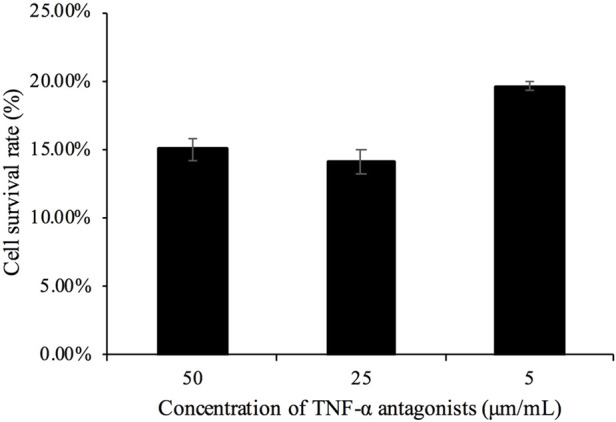
Concentration screening of TNF-α antagonists in inhibiting cell death. Note: Contains 1 μg/mL actinomycin D and 4 ng/mL TNF-α.

**FIGURE 7:**
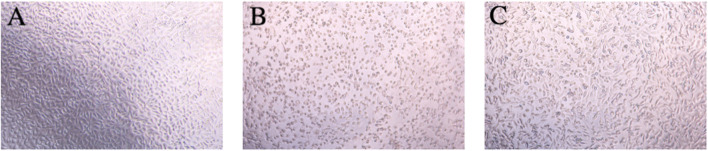
Cell morphology of L929. **(A)** Blank control group; **(B)** TNF-α combined with the actinomycin D stimulation group; **(C)** TNF-α antagonist treatment group.

It has been observed that the cell survival rate in the toxicity analysis of Actinomycin D combined with TNF-α on L929 cells was approximately 10% (Sun et al., 2020). As depicted in Figure 5, the TNF-α concentration of 4 ng/mL could reduce the cell survival rate to 12.9%, which was selected for subsequent experiments. As shown in Figure 6, increasing concentrations of the TNF-α antagonist were tested; the antagonist at 5 μm/mL was able to restore L929 cell viability in the presence of 4 ng/mL TNF-α (with ActD) to approximately 20%; thus, this dose was used for the morphological analysis (Figure 7).

As shown in Figure 7, the cellular morphological characteristics evolved dynamically, with a decrease in the cell density, an increase in the cytosolic contraction, and a significant shrinkage of cells with the increase in concentration. As shown in Figure 7B, the cells showed blurred outlines, reduced density, cytoplasmic shrinkage, and decreased pseudopodia, which is consistent with the pathological changes induced by TNF-α combined with apoptosis inducers. The observed cellular state in Figures 7A,B demonstrated that the antagonist interacts with TNF-α to mitigate its pro-apoptotic effect on cells.

L929 cells were markedly more sensitive to TNF-α than HepG2 cells, consistent with the known susceptibility of L929 to TNF-mediated cytotoxicity (Zhang et al., 2025).

### 3.4 AA-IDMS

#### 3.4.1 Validation of the correctness of AA-IDMS

CRM of BSA (NIM-RM3627-9) was used to determine its mass fraction, with Val, Leu, and Phe as the target amino acids. The corresponding results for BSA are presented in Table 2. The standard value was 2.1 ± 0.2 mg/g (*k = 2*). The degree of equivalence E_n_<1 was calculated according to Equation 3. Therefore, AA-IDMS was reliable and could be used for the quantification of TNF-α or peptides.

**TABLE 2:** Quantitative results of BSA standard substances.

Sample	Content (mg/g)			Average (mg/g)
1	1.974	1.971	1.968	1.971
2	1.966	1.966	1.965	1.966
3	1.956	1.967	1.956	1.960
Average (mg/g)	—	—	—	1.965
Relative standard deviation (%)	0.29%			

#### 3.4.2 Optimization of hydrolysis time and detection of TNF-α

**FIGURE 8:**
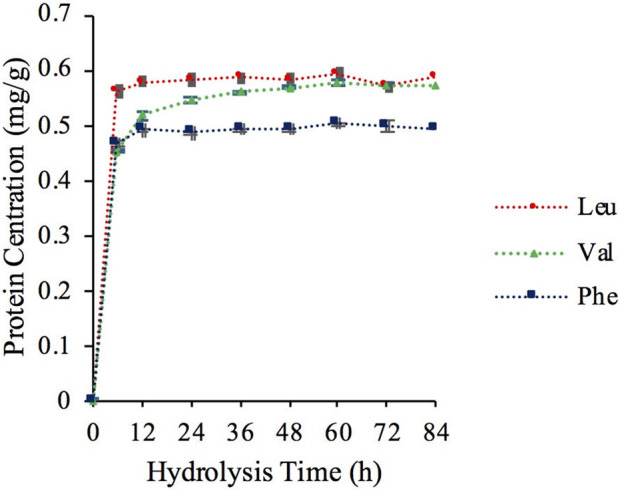
Detection results of TNF-α at different hydrolysis times.

According to the variation law of the response value ratio between target amino acids and isotope-labeled amino acids during mass spectrometry, hydrolysis was considered complete when the peak area ratio reached a maximum or tended to stabilize. This time point was thus identified as the optimal hydrolysis time. As shown in Figure 8, the peak area ratio of amino acids reached a stable plateau after 60 h of hydrolysis. Therefore, the optimal hydrolysis time of TNF-α was 60 h and is used for subsequent quantitative analysis by AA-IDMS.

Calculated according to Equations 1, 2, the results are shown in Table 3. The quantitative TNF-α content by AA-IDMS was 0.770 mg TNF-α per g of total solution.

**TABLE 3:** Quantitative results of TNF-α fixation based on amino acid analysis.

Sample	Content (mg/g)			Average (mg/g)
1	0.770	0.778	0.751	0.766
2	0.788	0.793	0.766	0.782
3	0.783	0.771	0.754	0.769
4	0.778	0.751	0.745	0.758
5	0.780	0.761	0.753	0.765
6	0.772	0.790	0.791	0.784
Average (mg/g)	—	—	—	0.770
Relative standard deviation (%)	1.23%			

### 3.5 PEP-IDMS

#### 3.5.1 AA-IDMS for characteristic peptides

The content of characteristic peptides was analyzed by Val and Leu. The specific quantitative results are shown in Table 4, and the contents of AR-12 and VK-9 in the target substance working solutions were measured to be 0.565 mg/g and 0.770 mg/g, respectively.

**TABLE 4:** Quantitative results of standard peptides.

Standard peptide	Characteristic peptide content (mg/g)		Average (mg/g)	RSD (%)
	Leucine	Valine		
ANALLANGVELR	0.557	0.571	0.565	0.59
VVNLLSAIK	0.747	0.794	0.770	1.80

#### 3.5.2 Optimization of enzymatic hydrolysis time and detection of TNF-α

**FIGURE 9:**
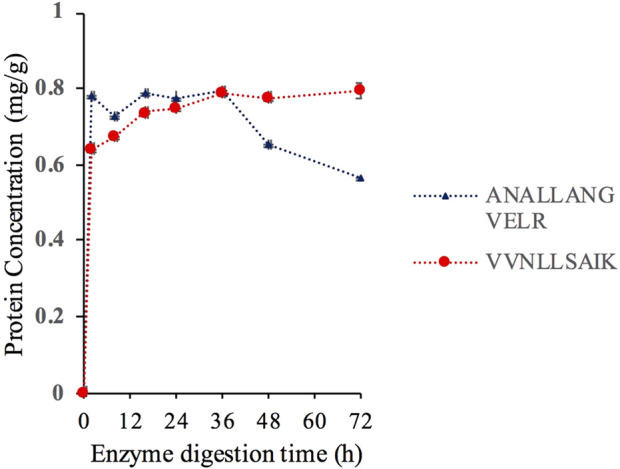
Results of TNF-α assay at different digestion times.

Enzymatic hydrolysis time significantly affected the results of proteolytic hydrolysis. Insufficient hydrolysis time resulted in incomplete hydrolysis, while excessive hydrolysis time reduced the experimental efficiency. The enzymatic hydrolysis efficiency and stability of the characteristic peptides were investigated at 2, 8, 16, 24, 36, 48, and 72 h of hydrolysis. The results are shown in Figure 9. The enzymatic hydrolysis curves of AR-12 and VK-9 reached their maximum enzymatic hydrolysis levels at 36 h of hydrolysis. Considering the enzymatic hydrolysis efficiency, stability, and response intensity of the selected peptides, the optimal enzymatic hydrolysis time was determined to be 36 h.

The content of the sample quantified based on characteristic peptides was 0.769 mg/g, as shown in Table 5. The equivalence of this assay with the results of amino acid analysis was calculated according to Equation 3. *E*_*n*_ *< 1* indicated that there was no significant difference between the results of AA-IDMS and PEP-IDMS, and these methods could be used for the quantification of TNF-α.

**TABLE 5:** TNF-α fixation results based on peptide analysis.

Sample	Content (mg/g)			Average (mg/g)
1	0.760	0.747	0.769	0.759
2	0.770	0.772	0.775	0.772
3	0.779	0.770	0.753	0.767
4	0.777	0.772	0.783	0.777
5	0.763	0.782	0.768	0.771
6	0.762	0.771	0.784	0.772
Average (mg/g)	—	—	—	0.769
RSD (%)	0.81%			

#### 3.5.3 Linearity

The results of the linearity of AR-12 and VK-9 are shown in Supplementary Table S2. A linear regression curve was constructed with the ratio of the peak areas of the characteristic peptides to their isotopes as the horizontal coordinate, with their mass ratios as the vertical coordinate. The results of PEP-IDMS for AR-12 and VK-9 showed a good linear relationship with *R* ^2^ values of 0.9997 and 0.9998, respectively.

#### 3.5.4 Precision test

The results of intra-day and inter-day precision are shown in Table 6. According to the methodological test data, RSDs of intra-day and inter-day precision were less than 3% for TNF-α samples, with low, medium and high concentration gradients. This demonstrates that the method exhibits excellent repeatability and reproducibility, meeting the validation requirements for bioanalytical methods.

**TABLE 6:** Intra-day and inter-day precision results.

Precision type	Mass fraction	Day	Content (mg/g)			CV(%)
Intra-day	High	1	0.76975	0.77193	0.77469	0.32
		2	0.76217	0.77058	0.78383	1.41
		3	0.79584	0.79498	0.78316	0.90
	Medium	1	0.07601	0.07473	0.07693	1.46
		2	0.07396	0.07527	0.07406	0.98
		3	0.07403	0.07816	0.07679	2.76
	Low	1	0.00777	0.00779	0.00778	0.13
		2	0.00771	0.00762	0.00784	1.43
		3	0.00795	0.00794	0.00783	0.84
Inter-day	High	​	0.73965	0.74061	0.75273	0.98
	Medium	​	0.07798	0.07873	0.07936	0.88
	Low	​	0.00790	0.00795	0.00782	0.83

#### 3.5.5 Consistency evaluation of the two methods

From Supplementary Table S3, it can be observed that the content of the characteristic peptides in the quantitative samples was 0.769 mg/g. A t-test was performed with the results based on the amino acid analysis, and the results are shown in Supplementary Table S3, with *p* = 0.820 > 0.05, indicating that there was no significant difference between the two methods.

#### 3.5.6 Uncertainty assessment of quantitative quantities

The sources of uncertainty mainly include the following:

Standard substances: the relative expanded uncertainty of the L-leucine purity standard substance was 0.4%, the relative expanded uncertainty of the L-phenylalanine purity standard substance was 0.4%, and the relative expanded uncertainty of the L-valine purity standard substance was 0.6%, with the expansion factor *k = 2*.

Uncertainty of balance weighing: the relative expanded uncertainty of the balance used was 0.04 mg, with the expansion factor *k = 2*.

Uncertainty introduced by hydrolysis efficiency: the uncertainty introduced by incomplete hydrolysis of TNF-α was estimated to be 1% according to the literature.

Uncertainty introduced by method repeatability: expressed by the standard deviation of the detection results.

The synthesis of the combined uncertainty from each uncertainty component is presented in Equation 5:

$$u_{c}=\sqrt{\sum_{i=1}^{n}{\left(\frac{\partial y}{\partial x_{i}}\times u_{i}\right)}^{2},}$$ (5)

where

*u*_*c*_, Combined standard uncertainty

*y*, The indirectly measured quantity

*x*_*i*_, The i-th directly measured input quantity

*n*, The number of input quantitiesy

*y*_*i*_. Standard uncertainty of the i-th input quantity xi

Calculation and synthesis of uncertainties were introduced using quantitative methods. Table 7 shows the calculation of uncertainty for Leu. After synthesizing the uncertainty of each influencing factor according to Equation 4, the uncertainty introduced by AA-IDMS and PEP-IDMS were determined to be 0.033 mg/g and 0.046 mg/g, respectively. Therefore, the concentration of TNF-α in the sample we used for method development was 0.770 ± 0.057 mg/g (*k = 2*).

**TABLE 7:** Calculation of quantitative uncertainty (leucine as an example).

Source of uncertainty	Uncertainty component	Sensitivity factor	Standardized uncertainty (mg/g)	Relative uncertainty (%)
Purity of reference standards	0.3	0.00513	0.00154	0.20
Sample weighing	0.02	0.00741	0.00015	0.01925
Internal standard working solution weighing	0.02	0.00741	0.00015	0.01925
Standard stock solution weighing (powder)	0.02	0.01911	0.00038	0.04964
Standard stock solution weighing (dilution)	0.02	0.00002	0.00000	0.00005
Standard working solution weighing (reserve solution)	0.02	0.00162	0.00003	0.00421
Standard working solution preparation (diluent)	0.02	0.00026	0.00001	0.00067
Hydrolysis efficiency	​		0.00770	1.00
Repeatable	​		0.00947	1.23
Synthetic standard uncertainty	​		0.01231	​
Expansion factors	​		2	​
Synthetic extended uncertainty	​		0.02462	​

## 4 Conclusion

Based on analytes with high purity, high activity, and accurate molecular weight, we have established the accurate, comparable, and traceable detection method for TNF-α. The AA-IDMS is more commonly used. After being fully hydrolyzed for 60 h at 110 °C, the content of TNF-α detected using AA-IDMS was 0.770 ± 0.033 mg/g (mg TNF-α per g of total solution, *k = 2*). PEP-IDMS based on AR-12 and VK-9 exhibits high specificity, effectively eliminating interference from sample impurities. The quantification of TNF-α was (0.769 ± 0.046) mg/g (*k = 2*) detected using PEP-IDMS after 36 h of tryptic proteolysis. The consistency between these two quantification methods was verified (*p* = 0.820 > 0.05). The concentration of TNF-α in the sample was 0.770 ± 0.057 mg/g (*k = 2*).

Due to the high specificity of the peptides, the isotope dilution method based on enzymatic digestion of characterized peptides may be more suitable for the determination of TNF-α in complex matrix samples. It ensured high specificity to avoid interference from miscellaneous proteins. It also resists matrix effects, retains structural information related to TNF-α activity, and offers higher sensitivity for low-abundance targets, with a feasible and efficient workflow for clinical batch analysis. For example, the bias values for trueness of PEP-IDMS used in serum C-peptide concentrations were <4%; however, the electrochemiluminescence immunoassay showed a positive bias of 51.8% (Cho et al., 2025). The method we established may improve the accuracy and reliability of TNF-α assay results and validate the comparability of the accuracy of the results of related assay kits on the market. As TNF-α remains a research hotspot, reliable measurement methods are crucial. Our study facilitates the evaluation of detection kits for this protein. However, the IDMS method has its own limitations, such as being labor-intensive and requiring further validation of its sensitivity in complex biological samples, which currently restrict its routine clinical application. Nevertheless, this work establishes a foundational reference method for the development of future certified reference materials.

## Data Availability

The original research results presented in this study are publicly available. These data can be obtained here: https://doi.org/10.6084/m9.figshare.31006972.
