# Supplementary figures and images for: Absolute quantification of tumor necrosis factor-alpha by isotope dilution mass spectrometry
Source: Front Chem. 2026 Feb 6;13:1667885. doi: 10.3389/fchem.2025.1667885 (PMC12921439; doi:10.3389/fchem.2025.1667885)

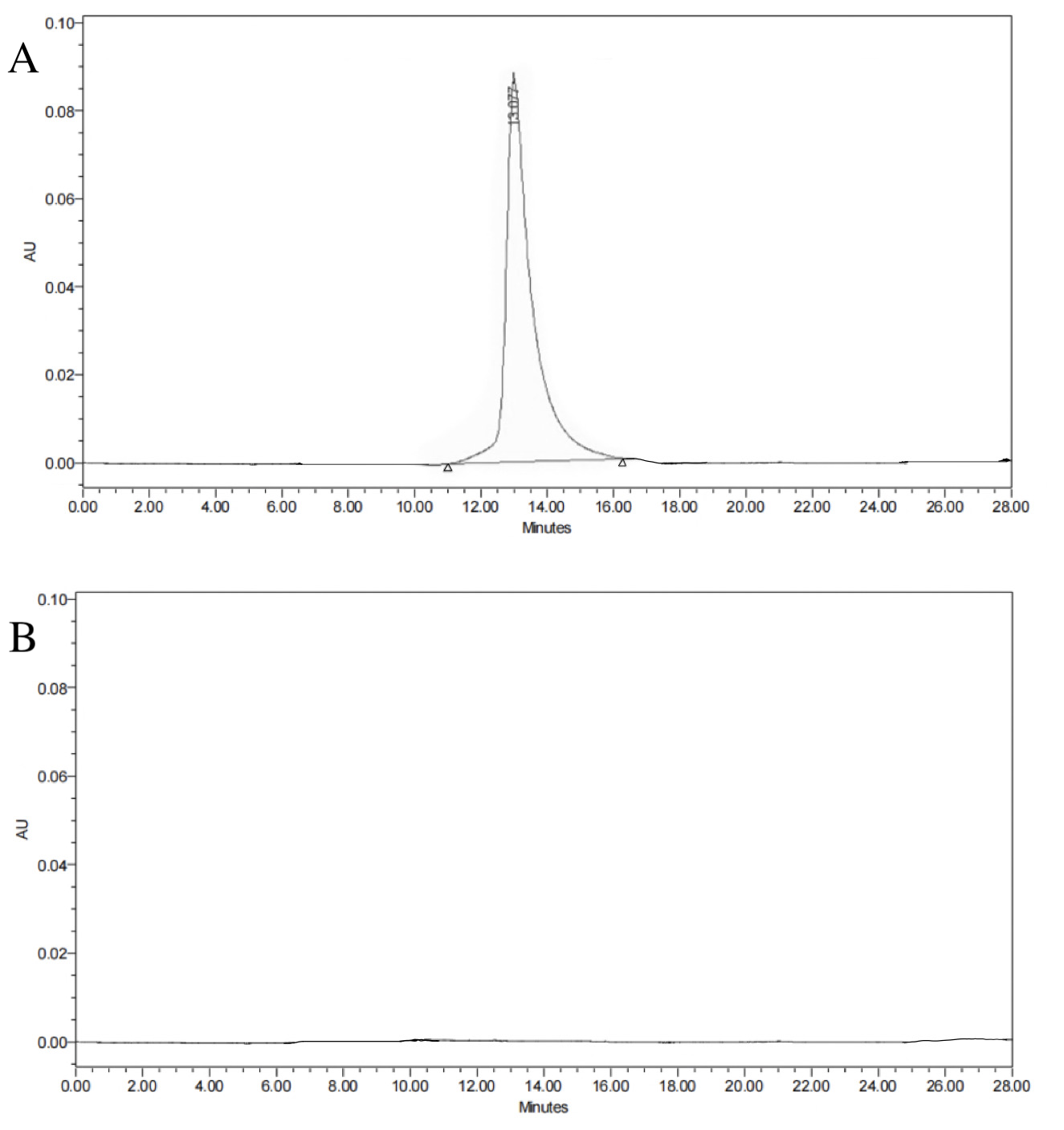

Supplement: Supplementary file 1 [file Image1.tiff]
