# Supplementary material for: Absolute quantification of tumor necrosis factor-alpha by isotope dilution mass spectrometry
Source: Front Chem. 2026 Feb 6;13:1667885. doi: 10.3389/fchem.2025.1667885 (PMC12921439; doi:10.3389/fchem.2025.1667885)
Supplement: Supplementary file 2 [file Table1.docx]

Supplementary Material

## Supplementary Tables

**Supplementary Table S1.** Repeatability evaluation of test kits.

| Samples | Magnetic particle chemiluminescence results (pg/mL) | | | Chemiluminescence results  (pg/mL) | | | | Flow fluorescence results  (pg/mL) | | | | |
| --- | --- | --- | --- | --- | --- | --- | --- | --- | --- | --- | --- | --- |
|  | Group 1 | Group 2 | Group 3 | Group 1 | Group 2 | Group 3 | Group 4 | Group 1 | Group 2 | Group 3 | Group 4 | Group 5 |
| 1 | 7.22 | 15.80 | 34.64 | 8.28 | 18.89 | 75.26 | 139.36 | 9.29 | 20.55 | 55.61 | 287.32 | 464.06 |
| 2 | 7.41 | 15.90 | 33.92 | 7.63 | 18.01 | 84.7 | 137.43 | 9.84 | 21.11 | 55.85 | 283.5 | 465.39 |
| 3 | 7.36 | 15.43 | 35.11 | 7.49 | 17.83 | 89.96 | 132.55 | 9.33 | 20.97 | 54.84 | 279.71 | 457.71 |
| Average(pg/mL) | 7.33 | 15.71 | 34.56 | 7.80 | 18.24 | 83.31 | 136.45 | 9.49 | 20.88 | 55.43 | 283.51 | 462.39 |
| RSD  (%) | 1.3% | 1.6% | 1.7% | 5.4% | 3.1% | 8.9% | 2.6% | 3.2% | 1.4% | 1.0% | 1.3% | 0.9% |
