# Supplementary material for: Absolute quantification of tumor necrosis factor-alpha by isotope dilution mass spectrometry
Source: Front Chem. 2026 Feb 6;13:1667885. doi: 10.3389/fchem.2025.1667885 (PMC12921439; doi:10.3389/fchem.2025.1667885)
Supplement: Supplementary file 6 [file Table2.docx]

Supplementary Material

## Supplementary Tables

**Supplementary Table S2.** Linear equations of mass ratio and peak area ratio of characteristic peptides to their isotopic internal standard.

| Characteristic Peptide | Linear Equation | Correlation Coefficient（R^2^） |
| --- | --- | --- |
| ANALLANGVELR | Y=0.8547x+0.0056 | 0.9997 |
| VVNLLSAIK | Y=1.2532x–0.0749 | 0.9998 |
