# Supplementary material for: Absolute quantification of tumor necrosis factor-alpha by isotope dilution mass spectrometry
Source: Front Chem. 2026 Feb 6;13:1667885. doi: 10.3389/fchem.2025.1667885 (PMC12921439; doi:10.3389/fchem.2025.1667885)
Supplement: Supplementary file 7 [file Table3.docx]

Supplementary Material

## Supplementary Tables

**Supplementary Table S3**. Comparison of accuracy based on peptides and amino acids.

| Average of peptide quantification results(mg/g) | RSD/% | Average of amino acid quantification results(mg/g) | RSD/% | p-Value |
| --- | --- | --- | --- | --- |
| 0.769 | 0.81% | 0.770 | 1.23% | 0.820 |
